# Supplying New Blood

**Published:** 1875-01

**Authors:** 


SUPPLYING NEW BLOOD.—It is clear that a man whose blood is reduced in quantity and quality can be supplied with an article from some of the lower animals which will answer an excellent purpose. Herman Dubois, of Fall River, Mass., had suffered from consumption for five years, and had become very weak and debilitated. Physicians advised him to seek a warmer climate, but he had not sufficient strength to avail himself of this chance of relief. Accordingly his physician determined to furnish him with a fresh supply of blood. A healthy, active lamb was procured and taken to the room where the patient reclined. The animal was laid upon its side. An incision was made on one side of the larynx, exposing the carotid artery. When this artery was fully exposed, a ligature was tied around the vessel, shutting off completely the blood current. At a distance of about an inch and a half below the ligature a powerful pair of forceps was applied to the artery, compressing the vessel perfectly. Thus there was a space between the ligature and the forceps which could be opened without danger of hemorrhage. A small incision was made into the artery in this inclosed space. Then a glass tube, slightly bent, was inserted into the artery. A small isthmus or constriction had been made in the part of the glass tube inserted into the artery, which enabled the tube to be securely tied into the vessel. After the tube had been secured in the lamb's artery, everything was ready for work upon the patient. In Mr. Dubois' arm the vein at the bend of his elbow connecting the basilic and cephalic veins was exposed. A bandage was tied around below the proposed incision to prevent a flow of venous blood from the wound. After exposing the vein by an incision an inch long, forceps were placed above and below, shutting off the blood current from a space about half an inch long. The lamb's neck was then brought close to the patient's arm, and the pressure of the forceps upon the lamb's artery relaxed. The blood rushed through the tube, expelling all the air. Then the opposite end was skillfully inserted into the patient's vein and the pressure of the forceps upon the lamb's artery removed. The bright blood leaped through the tube and entered the system of the patient. The stream was kept up for one minute and forty seconds. Then the tube was removed.

Mr. Dubois has sufficiently recovered his strength to enable him to visit a warmer climate with good prospects of regaining his health. The lamb is also alive and doing well. A lamb used in the same manner in a former experiment in this city, is still alive, and has never shown the least sign of having suffered from the experiment. The operation is a very simple one and can be performed by any educated medical man. It is moreover nearly painless, and no danger need be apprehended from it. The blood-corpuscles of the lamb mix readily with the human blood and quickly supply the loss induced by disease.

